# Impact of Stimulation Order on Clinical Outcomes in Bilateral iTBS Treatment

**DOI:** 10.1192/j.eurpsy.2026.11613

**Published:** 2026-07-31

**Authors:** T. T. Raij, M. Stenroos, D. B. Aydogan, T. Lehtilä, E. Komulainen

**Affiliations:** 1 University of Helsinki and Helsinki University Hospital, Helsinki; 2 Aalto University School of Science, Espoo; 3 University of Eastern Finland, Kuopio, Finland

## Abstract

**Introduction:**

Intermittent theta burst stimulation (iTBS) targeting the left dorsolateral prefrontal cortex (DLPFC) is a brief transcranial magnetic stimulation protocol widely used in treatment-resistant depression. Sequential bilateral stimulation—typically involving continuous TBS on the right DLPFC—has been proposed to improve efficacy, though findings remain inconsistent. A recent registry-based study suggested that initiating stimulation on the left side may yield better clinical outcomes, warranting further investigation.

**Objectives:**

This exploratory analysis aims to evaluate the effect of stimulation order in a recent controlled clinical trial.

**Methods:**

Patients were recruited from those referred to a university hospital neuromodulation unit for intermittent theta-burst stimulation (iTBS) treatment. We analyzed 78 patients with complete data: 39 randomized to standard left-sided iTBS and 39 to bilateral iTBS, where stimulation targets were individualized using fMRI connectivity of the dorsolateral prefrontal cortex (DLPFC) with the cingulo-opercular network and the subgenual cingulate cortex. Each patient received 20 to 25 treatment sessions. Treatment efficacy was measured by the percentage change in Montgomery–Åsberg Depression Rating Scale (MADRS) scores from baseline to post-treatment. Among the bilateral iTBS group, every other patient began each session with left-sided stimulation. The impact of stimulation order on clinical outcomes was evaluated using linear regression, adjusted for Maudsley treatment resistance. Baseline MADRS scores, sex, and number of sessions were included as covariates in a sensitivity analysis.

**Results:**

Patients who received bilateral stimulation starting with the left side showed a greater reduction in depression severity (mean +/- SD 46% +/- 29%, p = 0.23) compared to those who received standard left-sided stimulation (29% +/- 27%) or bilateral stimulation starting on the right side (28% +/- 28 %). The effect remained statistically significant in the sensitivity analysis (p < 0.009).

**Conclusions:**

These findings suggest that stimulation order may influence treatment outcomes in bilateral TMS. Larger, controlled studies are needed to confirm this effect and explore possible mechanisms related to hemispheric laterality, targeting, and priming. Such research could contribute to optimizing transcranial magnetic stimulation protocols.

**Disclosure of Interest:**

None Declared

